# Single-cell transcriptomics identifies an *H2AFZ*-driven proliferative tumor subpopulation associated with poor prognosis in hepatocellular carcinoma

**DOI:** 10.3389/fmolb.2025.1655705

**Published:** 2025-10-08

**Authors:** Jihui Huo, Tao Yang, Kai Lei, Zeyao Wang, Zebin Chen, Qi Zhou

**Affiliations:** ^1^ Department of Oncology, The First Affiliated Hospital of Sun Yat-sen University, Guangzhou, China; ^2^ Department of Hepatobiliary Surgery, Hui Ya Hospital of The First Affiliated Hospital, Sun Yat-sen University, Huizhou, China; ^3^ Department of Liver surgery, The First Affiliated Hospital of Sun Yat-sen University, Guangzhou, China; ^4^ Center of Hepato-Pancreato-Biliary Surgery, The First Affiliated Hospital of Sun Yat-sen University, Guangzhou, China

**Keywords:** hepatocellular carcinoma, prognostic signature, H2AFZ, single-cell RNA sequencing, tumor heterogeneity, tumor immune microenvironment

## Abstract

**Background:**

Hepatocellular carcinoma (HCC) is a highly heterogeneous cancer with complex tumor–immune interactions. This heterogeneity, particularly in tumor and immune cells, complicates treatment and prognostic evaluation. Although recent studies have revealed distinct tumor cell states and immune dysfunction in HCC, the molecular basis underlying tumor aggressiveness remains poorly understood. A deeper understanding of the molecular and functional diversity of both tumor and immune cell populations, especially the identification of stem-like tumor subpopulations and immunosuppressive mechanisms, along with the development of robust prognostic biomarkers, is essential for advancing precision oncology and improving clinical outcomes.

**Methods:**

We integrated three publicly available single-cell RNA sequencing (scRNA-seq) datasets from GEO to delineate the cellular architecture of the HCC tumor microenvironment. Unsupervised clustering and dimensionality reduction were employed to identify major cell types and tumor subpopulations. Functional annotation was performed using canonical markers, Monocle, CytoTRACE, and AUCell scoring. *H2AFZ* was identified as a candidate oncogene and validated through *in vitro* knockdown experiments. The interaction between T cell subsets and tumor subpopulations were further characterized. A prognostic risk model was constructed using LASSO regression.

**Results:**

Six major cell types were identified in HCC TME. Tumor cells were subdivided into three distinct clusters: Tumor_C0, Tumor_C1 and Tumor_C2. Tumor_C2 showed the highest stemness, pro-metastatic activity and immunogenic cell death signatures. *H2AFZ* was highly expressed in Tumor_C2 and associated with poor prognosis. The knockdown of *H2AFZ* reduced H2A.Z protein levels, inhibited proliferation, invasion, and induced apoptosis. T cell analysis revealed five subpopulations. It was found that Tumor_C2 interacts with the proliferative and exhausted T cell subpopulations, suggesting a potential functional relationship between them. The prognostic model based on tumor_C2 transcriptomic features effectively stratified patient survival across multiple cohorts, with robust AUCs and Kaplan-Meier survival distinctions.

**Conclusion:**

We identified a proliferative, stem-like tumor cell subpopulation (Tumor_C2) in HCC characterized by high *H2AFZ* expression, which drives tumor aggressiveness. T cell analysis revealed significant interactions with Tumor_C2. Moreover, a prognostic model based on Tumor_C2 features effectively stratified patient survival across multiple cohorts. Together, these findings highlight potential therapeutic targets for improving patient outcomes.

## 1 Introduction

HCC, the most prevalent primary liver malignancy, accounts for nearly half of global cases in China (Bray et al., 2024). The disease can be driven by a variety of etiological factors, including chronic hepatitis B or C virus (HBV/HCV) infections, non-alcoholic steatohepatitis (NASH), and chronic alcohol abuse (Global Burden of Disease Liver Cancer Collaboration et al., 2017; Llovet et al., 2021). Radical treatments for early-stage HCC primarily include total or partial hepatectomy and liver transplantation. For patients with intermediate-stage HCC, locoregional therapies such as transarterial chemoembolization (TACE) are considered the preferred approach. In advanced-stage HCC, systemic therapy remains the only option to provide a survival benefit (Reig et al., 2022). However, its five-year survival remains dismal (∼12.1%) due to frequent late-stage diagnosis (Bray et al., 2024).

Previous studies have demonstrated significant molecular complexity and heterogeneity in HCC, encompassing both intertumoral and intratumoral diversity, which pose substantial challenges to elucidating the mechanisms underlying tumor progression (Caruso et al., 2019; Huang et al., 2017). This heterogeneity partly accounts for the observed variability in patient survival outcomes and therapeutic responses (Llovet et al., 2016; Llovet et al., 2021; Peng et al., 2025). Moreover, specific tumor cell subpopulations exhibit malignant phenotypes characterized by enhanced proliferative capacity and multilineage differentiation potential (Zheng et al., 2018). Therefore, comprehensive characterization of tumor cell subpopulations in terms of their molecular profiles and functional states is critical for advancing biomarker discovery and optimizing personalized therapeutic strategies.

The interplay between tumor cells and components of the tumor immune microenvironment (TIME) plays a pivotal role in tumor progression and markedly influences therapeutic efficacy (Dunn et al., 2002). TIME represents a complex and dynamic ecosystem composed of various cell types, among which immune cells are central regulators of tumor progression and treatment response (Binnewies et al., 2018; Xu et al., 2020). Immune cells, including T lymphocytes, natural killer (NK) cells, macrophages, dendritic cells, and myeloid-derived suppressor cells (MDSCs), exhibit extensive phenotypic and functional heterogeneity. The application of single-cell sequencing technologies has substantially enhanced our ability to dissect this cellular diversity at unprecedented resolution, enabling precise identification of distinct immune subpopulations and their activation states within tumors (Tirosh et al., 2016; Savas et al., 2018). A deeper understanding of the intricate crosstalk between tumor cells and immune cells is crucial for elucidating mechanisms of tumor immune evasion, refining immunotherapeutic approaches, and predicting clinical outcomes (Binnewies et al., 2018; Joyce and Fearon, 2015). However, the specific mechanisms underlying tumor-immune interactions in HCC remain incompletely understood.

In this study, we performed scRNA-seq analysis on HCC samples and identified a proliferative tumor cell subpopulation, designated tumor_C2, characterized by marked proliferative activity and stemness features, as well as association with immunogenic cell death. We found that *H2AFZ* was significantly upregulated in tumor_C2, its expression positively correlated with the proliferative capacity of HCC cells *in vitro* and negatively correlated with patient survival. Cell-cell communication analysis revealed strong interactions between tumor_C2 and both C1_Cytotoxic (cytotoxic T cells) and C3_Cycling (proliferative T cells) subsets. Furthermore, the gene signature of tumor_C2 demonstrated prognostic value in predicting patient outcomes. Collectively, these findings enhance our understanding of tumor heterogeneity and immune interactions in HCC, suggesting that tumor_C2 may serve as a valuable biomarker for patient stratification and immunotherapy optimization, while *H2AFZ* may represent a candidate therapeutic target.

## 2 Materials and methods

### 2.1 Ethics statement

All human transcriptomic data analyzed in this study were obtained from publicly available, de-identified datasets. Bulk RNA-seq and clinical data for hepatocellular carcinoma (HCC) patients were retrieved from the TCGA-LIHC cohort via the UCSC Xena platform (https://xenabrowser.net), single-cell RNA-seq data GSE151530, GSE125449, and GSE149614 were obtained from Gene Expression Omnibus (GEO) datasets. The original studies generating these datasets received appropriate ethical approval from their respective institution (Ma et al., 2019; Wang et al., 2025; Lu et al., 2022; Jiang et al., 2024). No new human participants were recruited, and no identifiable private information was accessed.

### 2.2 Data collection

Bulk transcriptome and clinical data for HCC patients were obtained from the UCSC Xena platform (https://xenabrowser.net), specifically from TCGA liver hepatocellular carcinoma (LIHC) cohort. To maintain data integrity, samples lacking survival information, those with a survival time of zero, and biological or technical duplicates were excluded. A total of 363 high-quality tumor samples with complete clinical annotations were retained for downstream analyses. For external validation, the microarray dataset GSE14520, along with corresponding clinical metadata, was retrieved from the Gene Expression Omnibus (GEO) repository.

### 2.3 scRNA-seq data preprocessing

Single - cell transcriptomic datasets were retrieved from the GEO database, including GSE151530, GSE125449, and GSE149614, which comprised scRNA - seq profiles of HCC tumors. In total, data from 28 HCC patients who had undergone surgical resection for primary and previously untreated liver tumors were collected from these three datasets, with GSE151530 containing data from 13 patients, GSE125449 from 5 patients, and GSE149614 from 10 patients. The inclusion criteria for patients were: being diagnosed with HCC, having undergone surgical resection for primary liver tumors, and having no prior treatment for the liver tumor before sample collection. Information regarding the number of cells per sample and other relevant details are provided in Table 1. Raw gene expression matrices based on unique molecular identifiers (UMIs) were imported into the R software environment (v4.3.1) and processed using the “Seurat” package (v4.3.0.1). Before filtering individual cells, samples with fewer than 500 cells in total were excluded from the analysis, meaning that each individual sample must have at least 500 cells. Cells were further filtered based on the following criteria: expression of fewer than 300 genes (nFeature_RNA), greater than 15% mitochondrial gene content (percent.mt), and a total RNA count exceeding 10,000 UMIs (nCount_RNA). After applying these QC filters, data were normalized using the NormalizeData function and scaled using ScaleData. Variable features were identified using FindVariableFeatures, and dimensionality reduction was performed via principal component analysis (PCA) and UMAP. To minimize the impact of doublets, the DoubletFinder (v2.0.3) algorithm was applied, with the expected doublet rate estimated using a Poisson distribution model based on the cellular density. The expected doublet rate was set to 7.5%, and the doublet classification was refined using optimized parameters (pN = 0.25, pK chosen based on the Bayesian Information Criterion, and nExp adjusted for homotypic doublets). Singlet cells were retained for downstream analysis, and results were stored in the metadata. Following rigorous quality control, a total of 41,301 single cells across 25,714 expressed genes were included for integrative downstream analyses.

**TABLE 1:** Overview of single - cell RNA sequencing data from HCC patients. This table presents the dataset cohort information, patient identification, the number of cells, and the number of genes for each sample in the study. The datasets were obtained from the GEO database, including GSE151530, GSE125449, and GSE149614, and the samples were collected from HCC patients who underwent surgical resection for primary and previously untreated liver tumors.

Dataset cohort	Patient ID	Number of cells
GSE151530	Patient01	1,155
GSE151530	Patient02	2,550
GSE151530	Patient03	1,674
GSE151530	Patient04	997
GSE151530	Patient05	1,243
GSE151530	Patient06	475
GSE151530	Patient07	3,333
GSE151530	Patient08	1,824
GSE151530	Patient09	2,186
GSE151530	Patient10	428
GSE151530	Patient11	2,118
GSE151530	Patient12	1,230
GSE151530	Patient13	2,476
GSE125449	Patient14	579
GSE125449	Patient15	765
GSE125449	Patient16	379
GSE125449	Patient17	610
GSE125449	Patient18	579
GSE149614	Patient19	2,232
GSE149614	Patient20	3,152
GSE149614	Patient21	1,655
GSE149614	Patient22	478
GSE149614	Patient23	1,357
GSE149614	Patient24	2,568
GSE149614	Patient25	1,734
GSE149614	Patient26	1,931
GSE149614	Patient27	373
GSE149614	Patient28	3,348

### 2.4 Inference of CNVs based on scRNA-seq

To identify malignant cells, we applied inferCNV analysis (v1.16.0, https://github.com/broadinstitute/inferCNV) using T cells as reference populations to infer chromosomal copy number variation (CNV) patterns from the filtered raw UMI counts (cutoff = 0.1, denoise = TRUE, HMM = F).

### 2.5 Developmental trajectory inference

To explore the developmental dynamics and functional heterogeneity of tumor cell subsets, we performed pseudotime analysis using two complementary tools: Monocle3 (v2.1.2) and CytoTRACE2 (v1.0.0). In Monocle3, highly variable genes were selected based on unsupervised clustering, and dimensionality reduction was carried out using PCA and UMAP. Cells were then ordered along a reconstructed trajectory by defining a biologically relevant root node.

For independent validation, following the standard pipeline, we applied the *CytoTRACE2::run_CytoTRACE2()* function, which integrates gene expression entropy and known transcriptional programs to generate a pseudotime-like ordering of cells (Kang et al., 2024).

### 2.6 Tumor cell, T-cell dimensionality reduction, clustering, and annotation

For data integration across the three single-cell RNA-sequencing datasets, we employed the Harmony (v1.2.0) algorithm to correct for batch effects and ensure robust downstream analysis (Korsunsky et al., 2019). Both tumor cells and T-cells were extracted and processed separately using Seurat pipeline.

For tumor cells, the data were normalized using the NormalizeData function, highly variable features were identified, and principal component analysis (PCA) was performed. Harmony integration was applied to mitigate dataset-specific batch effects while preserving biological heterogeneity. Following dimensionality reduction, clustering analysis was performed using the FindClusters function, resulting in the identification of three distinct tumor cell clusters. UMAP visualization was used to display the integrated tumor cell populations.

Similarly, T-cells underwent the same preprocessing and integration workflow. After Harmony-based batch correction, clustering analysis identified five distinct T-cell subpopulations. t-SNE was employed for visualization of T-cell clusters. Cluster annotations were assigned based on canonical functional gene markers: C0_Naive (IL7R, LTB), C1_Cytotoxic (GZMH, GZMB), C2_Exhaustion (CTLA4, BATF), C3_Cycling (MKI67, STMN1), and C4_Tfh (CXCL13, CD200).

### 2.7 Cellchat analysis

To analyze intercellular communication between distinct tumor cell populations and T cell subsets, we utilized the CellChat R package (v2.1.2) with a customized approach. After preprocessing the single-cell transcriptomic data, we initialized the analysis by building separate CellChat objects for each tumor cell group using the createCellChat constructor. Instead of directly computing all possible communications, we implemented a stepwise filtering strategy to enhance specificity: interactions involving fewer than ten cells in either interacting cell type were excluded prior to downstream modeling. Communication probability matrices were then inferred via the *computeCommunProb* function. This pipeline ensured a stringent identification of relevant ligand-receptor interactions while minimizing noise from underrepresented cell populations.

### 2.8 Analysis of *H2AFZ* expression and prognostic relevance

We analyzed the mRNA expression level of *H2AFZ* and its association with overall survival (OS) in HCC using the GEPIA2 web tool (http://gepia2.cancer-pku.cn), which integrates RNA-sequencing data from the TCGA-LIHC and GTEx datasets. Differential expression analysis was performed to compare *H2AFZ* levels between tumor and normal liver tissues. For survival analysis, patients were stratified into high- and low-expression groups based on the median expression value of *H2AFZ*. Kaplan–Meier survival curves were generated through the “Survival Analysis” module, and statistical significance was assessed using the log-rank test.

### 2.9 Cross-platform data harmonization and batch effect correction

To ensure robust cross-platform validation between RNA-seq (TCGA-LIHC) and microarray (GSE14520) datasets, we implemented a comprehensive data harmonization pipeline. First, gene mapping was performed by identifying common genes present in both platforms using the *intersect* function in R, ensuring consistent gene annotation across datasets. Expression matrices were subset to include only these common genes, with identical gene ordering maintained between platforms.

For batch effect correction, we employed the ComBat algorithm implemented in the sva R package (v3.48.0). The expression matrices from both platforms were merged, with batch labels assigned to distinguish TCGA and GEO samples. The *ComBat* function was applied with *par. prior = TRUE* parameter to utilize parametric empirical Bayes adjustments. This method effectively removes platform-specific technical variations while preserving biological signal integrity. The corrected expression data were then separated back into individual platform datasets for subsequent analysis.

### 2.10 Prognostic model construction and validation

To evaluate the prognostic risk scoring model (STRS) based on tumor_C2-associated genes in HCC patients, we employed the following methodology. The Least Absolute Shrinkage and Selection Operator (LASSO) regression was then performed using the “glmnet” R package (Friedman et al., 2010) to refine the tumor_C2 gene set and build a prognostic model within a Cox proportional hazards framework. Each patient’s STRS was calculated. Patients were stratified into high- and low-risk groups based on the median STRS. Kaplan-Meier survival analysis was conducted using the “survival” (v3.5-7) and “survminer” (v0.4.9) R packages to assess the association between STRS and overall survival (OS). A *p*-value of less than 0.05 was considered statistically significant. Receiver operating characteristic (ROC) curves were also generated to evaluate the predictive performance of the STRS model.

### 2.11 Cell culture

The human HCC cell line SNU-449 (RRID: CVCL_0454) was obtained from the Cell Bank of the Chinese Academy of Sciences (Shanghai, China), authenticated by short tandem repeat (STR) profiling, confirmed to be free of *mycoplasma* contamination using PCR-based testing within 6 months of use. Cells were maintained in Dulbecco’s Modified Eagle Medium (Procell) supplemented with 10% fetal bovine serum (Procell) and 1% penicillin-streptomycin (Gibco). Cells were maintained at 37 °C in a humidified incubator with 5% CO_2_.

### 2.12 RNA silencing

*H2AFZ* short interfering RNAs (siRNAs) targeting two different sequences (siRNA-1: GCGACUGCCGCUGUGUACATT; siRNA-2: CGAGUCUUAACCAUAUUUATT) and a negative control siRNA (UUCUCCGAACGUGUCACGU) were synthesized and used at a final concentration of 10 nM. Transfections were performed using Lipofectamine RNAiMAX (Invitrogen, 13778100) following the manufacturer’s instructions. Briefly, HCC cells were seeded in 6-well plates at a density of 2 × 10^5^ cells per well and incubated at 37 °C with 5% CO_2_ for 24 h prior to transfection. For each well, siRNA and RNAiMAX reagent were separately diluted in Opti-MEM medium (ThermoFisher Scientific, 31985070) and then combined to form siRNA-lipid complexes. After 15 min of incubation at room temperature, the complexes were added dropwise to the cells. Transfected cells were harvested 48 h later for assessing knockdown efficiency through Western blot analysis and downstream assays, including proliferation, migration, invasion, and gene expression analyses.

### 2.13 Western blotting

Cells were lysed using RIPA buffer supplemented with 1% phosphatase and protease inhibitors (Sigma). Protein concentration was measured using a BCA protein assay kit (Epizyme). 20–40 μg aliquot of protein lysate was resuspended in Omni-Easy^TM^ Protein Sample Loading Buffer (Denaturing, Reducing, 5×) (Epizyme) and denatured at 95 °C for 10 min. Proteins were separated by electrophoresis on 15% PAGE gels (Epizyme) using Tris-glycine (Epizyme) (80–100 V for 2 h), followed by transfer to activated PVDF membranes (Merck Millipore) at 100 V for 40 min. The membranes were then incubated in protein-free rapid blocking buffer (Epizyme) at room temperature for 1 h, followed by overnight incubation with anti-Histone H2A.Z antibody at 4 °C and 1-h incubation with HRP-conjugated secondary antibodies at room temperature. After washing three times (10 min each) with PBS containing 0.1% Tween-20, antibody signals were detected using the Omni-ECL^TM^ ultrasensitive chemiluminescence detection kit (Epizyme), and target proteins were visualized using the ImageQuant 800 imaging system (GE Healthcare).

### 2.14 Colony proliferation and formation assay

For proliferation assays, 1 × 10^3^ cells per well were seeded onto a 96-well plate and use the Cell Counting Kit-8 (CCK-8) cell proliferation and cytotoxicity assay kit (DOJINDO LABORATORIES) to measure cell proliferation. Add CCK8 to the culture medium, incubate in the dark at 37 °C for 2 h, then measure the absorbance at 450 nm using a multifunctional microplate reader (Thermofisher).

For formation assays, 1 × 10^3^ cells per well were seeded onto a 6-well plate and replace the culture medium every 3 days. After 10 days of treatment, wash once with PBS, fix the colonies with 4% paraformaldehyde for 15 min, rinse with water, and stain with crystal violet for 5–10 min. Quantify the number of cell colonies using ImageJ software. The experiment was conducted with triplicates per group.

### 2.15 Cell apoptosis assays

Cells were collected and washed twice with PBS, then resuspended in PBS after centrifugation. Apoptosis was detected using the Annexin V, FITC Apoptosis Detection Kit (DOJINDO LABORATORIES). All stained cells were analyzed using the Attune NxT Flow Cytometer (Thermo Fisher). Data was processed with FlowJo software.

### 2.16 Cell migration and invasion assay

For migration assays, 24-well transwell inserts (Falcon®) were placed into wells containing 700 μL of medium supplemented with serum. A total of 1 × 10^5^ cells suspended in 200 μL of serum-free medium were added to the upper chamber. Cells were incubated at 37 °C for 48–72 h to allow migration. At the endpoint, non-migrated cells on the upper membrane surface were removed gently. The inserts were washed with PBS, fixed in 4% paraformaldehyde for 15 min, and stained with 0.5% crystal violet for 10 min. After final rinsing, migrated cells on the lower membrane surface were imaged using an Olympus IX83 automated inverted microscope, and quantified using GraphPad Prism.

Cell invasion was assessed using Matrigel-coated transwell inserts. The upper surface of each insert was pre-coated with Matrigel matrix. Next, 1 × 10^5^ cells in 500 μL of serum-free medium were seeded into the upper chamber, while the lower chamber contained medium with serum to serve as a chemoattractant. After 72 h of incubation at 37 °C, non-invading cells were removed, and invaded cells on the underside of the membrane were fixed and stained with 0.5% crystal violet for 10 min. The number of invasive cells was counted under Olympus IX83 to evaluate invasion capacity.

### 2.17 Statistical analysis

Statistical analysis was performed using R software (v4.3.1). Categorical variables were compared using either the Chi-square test or Fisher’s exact test, as appropriate. For continuous variables, the Student’s t-test or Wilcoxon rank-sum test was employed based on data distribution. *p* < 0.05 was considered indicative of statistical significance. Effect sizes were calculated and reported alongside p-values: Cohen’s d for t-tests, r for Wilcoxon tests, hazard ratios (HR) for survival analyses, and n^2^ for ANOVA. Confidence intervals (95% CI) were calculated for all effect estimates. Multiple testing corrections were applied to control the false discovery rate (FDR) across different analyses. For differential expression analysis, the Benjamini-Hochberg procedure was used to control FDR at α = 0.05, with adjusted *p*-values (q-values) reported. For pathway enrichment and AUCell score comparisons involving multiple gene sets, FDR correction was applied using the Benjamini-Hochberg method. For pairwise comparisons in functional assays, Bonferroni correction was applied. All corrected *p*-values (*q*-values) are reported alongside effect sizes and 95% confidence intervals. *P*-values were reported in standardized format: values ≥0.001 as *p* = 0. xxx, and values < 0.001 in scientific notation as *p* = x.xx×10^−n^.

## 3 Result

### 3.1 Single-cell transcriptomics maps the cellular landscape of the HCC tumor microenvironment

**FIGURE 1:**
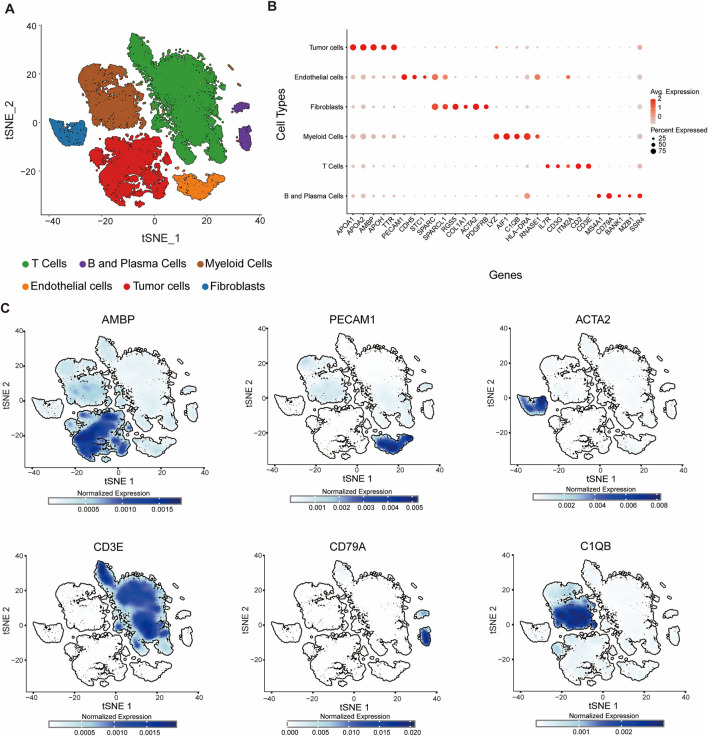
Single-cell transcriptomic profiling reveals cellular diversity within the tumor microenvironment. **(A)** t-SNE plot showing six distinct cell clusters identified by unsupervised clustering of single-cell RNA-seq data. Each cluster is annotated based on canonical marker gene expression: T cells (green), B and plasma cells (purple), myeloid cells (brown), endothelial cells (orange), tumor cells (red), and fibroblasts (blue). **(B)** Bubble plot showing lineage-specific gene expression across cell types in the tumor microenvironment. Each row represents a distinct cell population (B and Plasma Cells, T Cells, Myeloid Cells, Fibroblasts, Endothelial Cells, Tumor Cells), and each column represents a specific gene. The color intensity indicates the average expression level (red: high, light gray: low), while the size of the bubble reflects the percentage of cells expressing the gene within each population. **(C)** Feature plots of normalized expression for individual marker genes projected onto the t-SNE space: AMBP, PECAM1, ACTA2, CD3E, CD79A, C1QB. Marker expression is enriched in corresponding cell clusters, validating cell type annotation.

To characterize the cellular architecture within the tumor microenvironment of HCC, we integrated three publicly available single-cell RNA-sequencing datasets (GSE151530, GSE125449, and GSE149614) obtained from the GEO. We performed single-cell transcriptomic analysis followed by t-SNE for dimensionality reduction on the integrated dataset, which revealed that cells from 28 patients were evenly distributed across clusters (Supplementary Figure S1A), supporting the robustness of our integration strategy. Unsupervised clustering identified six major cell populations: T cells, B and plasma cells, myeloid cells, endothelial cells, fibroblasts, and tumor cells (Figure 1A). The respective cell counts for each population are as follows: T cells (18,679), B and plasma cells (1,446), myeloid cells (8,549), endothelial cells (2,488), fibroblasts (1,969), and tumor cells (10,301).

Similar to previous studies, cell populations were annotated based on the expression of canonical lineage markers (Sun et al., 2021). To comprehensively characterize cellular heterogeneity within the tumor microenvironment, we systematically analyzed the expression patterns of 29 key lineage-specific genes across distinct cell populations and visualized them using bubble plots (Figure 1B). The analysis revealed selective enrichment of specific markers in their respective cell types: MS4A1, CD79A, BANK1, MZB1, and SSR4 in B and plasma cells; IL7R, CD3G, ITM2A, CD2, and CD3E in T cells; LYZ, AIF1, C1QB, HLA-DRA, and RNASE1 in myeloid cells; RGS5, COL1A1, ACTA2, and PDGFRB in fibroblasts; PECAM1, CDH5, STC1, SPARC, and SPARCL1 in endothelial cells; and APOA1, APOA2, AMBP, APOH, and TTR in tumor cells (Figures 1B,C). The detection of these lineage-specific genes not only validated the identity of each cell population but also highlighted the heterogeneity within specific compartments, such as plasma cell-associated genes in B/plasma cells and tumor-specific markers in tumor cells. We also applied inferCNV (https://github.com/broadinstitute/inferCNV) using T cells as reference populations to infer chromosomal copy number variation (CNV) patterns. By calculating the CNV intensity score of each cell from the denoised matrix and integrating the HMM predictions, tumor cells with significantly higher CNV intensities compared to the reference, supporting the validity of classification (Supplementary Figure S1B). Collectively, these data delineate the cellular landscape of the tumor microenvironment and provide a clear, quantitative visualization of marker gene specificity, reinforcing the reliability of cell type annotations based on established lineage markers.

### 3.2 Three subtypes in HCC tumor cell heterogeneity landscape

**FIGURE 2:**
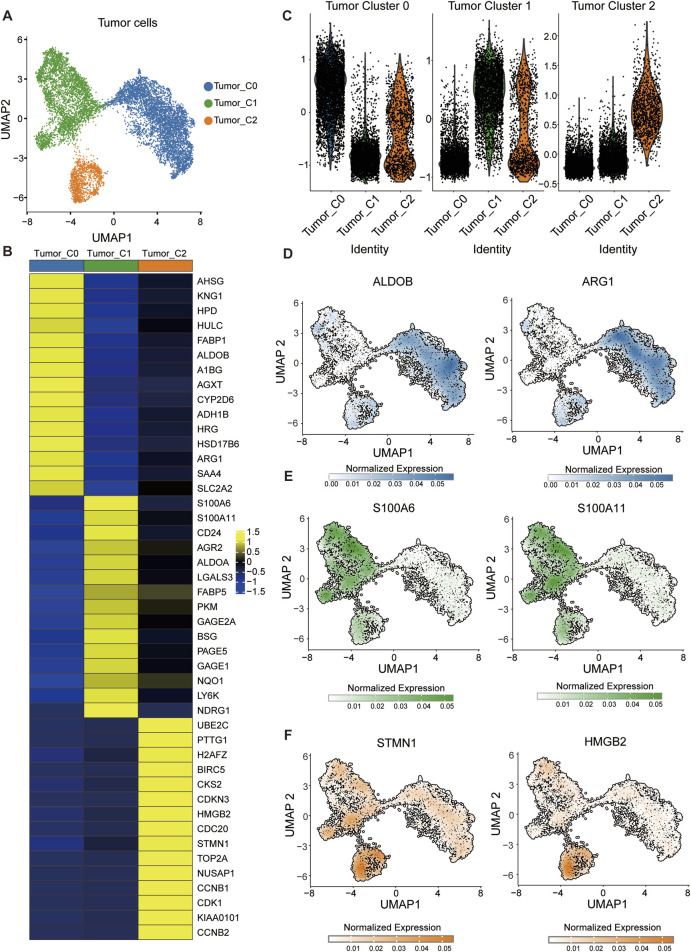
Identification of transcriptionally distinct tumor cell subpopulations in HCC. **(A)** UMAP visualization of tumor cells showing three distinct clusters: Tumor_C0 (blue), Tumor_C1 (green), and Tumor_C2 (orange). **(B)** Heatmap displaying differentially expressed genes across the three tumor cell clusters. (Wilcoxon rank-sum test; adjusted *p* < 0.05, log2FC > 0.5; FDR corrected using Benjamini-Hochberg procedure) **(C)** Module score analysis of tumor cell clusters. Violin plots showing the distribution of module scores calculated using AddModuleScore function for three distinct tumor cell populations. **(D–F)** Feature plots showing normalized expression of representative markers for each tumor cluster: **(D)** ALDOB and ARG1 (Tumor_C0), **(E)** S100A6 and S100A11 (Tumor_C1), and **(F)** STMN1 and HMGB2 (Tumor_C2).

To investigate intratumoral heterogeneity within HCC, we focused on tumor cell subsets extracted from the integrated single-cell transcriptomic dataset. Unsupervised clustering and UMAP embedding revealed three transcriptionally distinct tumor cell populations, designated as Tumor_C0, Tumor_C1, and Tumor_C2 (Figure 2A). Differential expression analysis identified unique gene signatures associated with each cluster (Figure 2B; Supplementary Figure S1C). To quantitatively assess the molecular signatures of these tumor clusters, we employed the AddModuleScore function in Seurat to calculate signature scores for each cluster using specific gene panels (Figure 2C). Consistent with previous findings (Guo et al., 2024), Tumor_C0 exhibited high expression of metabolism-related genes including ARG1 and ALDOA, suggesting preserved hepatocyte metabolic functions and representing a metabolically active tumor phenotype; Tumor_C1 was characterized by elevated expression of S100A6 and S100A11, which actively promote tumor progression and metastasis through calcium-binding signaling pathways and epithelial-mesenchymal transition processes; Tumor_C2 predominantly expressed proliferation-associated genes, notably TOP2A and STMN1, indicating high proliferative activity and cell cycle progression, which is typically associated with aggressive tumor behavior and poor prognosis in hepatocellular carcinoma (Figures 2D–F). Using the FindMarkers function in Seurat with Wilcoxon rank-sum test, we identified cluster-specific marker genes (adjusted *p*-value <0.05, log2FC > 0.5, FDR correction using Benjamini-Hochberg procedure). Tumor_C0 exhibited high expression of metabolic genes such as *ALDOB* and *ARG1* (Figure 2C), indicative of hepatocyte-like differentiation. In contrast, Tumor_C1 was enriched for *S100A6* and *S100A11* (Figure 2D), markers linked to Epithelial–Mesenchymal Transition (EMT). Tumor_C2 expressed proliferation and cell cycle–related genes including *STMN1* and *HMGB2*, suggestive of a highly proliferative phenotype (Figure 2E).

### 3.3 Developmental hierarchy and functional states of HCC tumor subpopulations

**FIGURE 3:**
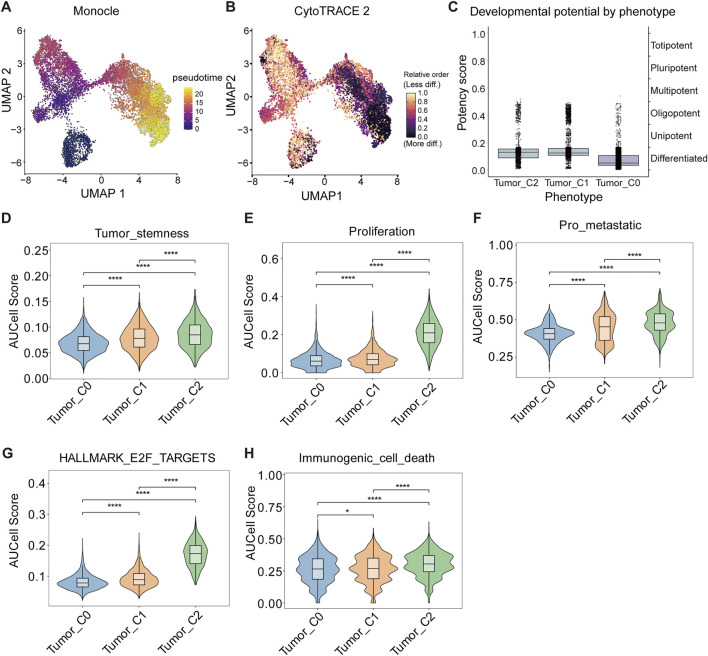
Identification of a stem-like and proliferative tumor cell subpopulation in HCC. **(A)** Pseudotime trajectory of malignant cells inferred using Monocle, colored by pseudotime rank. **(B)** Cell state differentiation inferred by CytoTRACE, colored by predicted developmental potential (less to more differentiated). **(C)** Box plots comparing CytoTRACE-defined developmental potency scores across tumor cell phenotypes (Tumor_C0–C2). **(D–F)** AUCell scores showing enrichment of stemness (Effect sizes: Tumor_C0 vs. Tumor_C1: r = 0.215, Tumor_C0 vs. Tumor_C2: r = 0.265, Tumor_C1 vs. Tumor_C2: r = 0.104) **(D)**, proliferation (Effect sizes: Tumor_C0 vs. Tumor_C1: r = 0.099, Tumor_C0 vs. Tumor_C2: r = 0.62, Tumor_C1 vs. Tumor_C2: r = 0.646) **(E)**, and pro-metastatic (Effect sizes: Tumor_C0 vs. Tumor_C1: r = 0.213, Tumor_C0 vs. Tumor_C2: r = 0.368, Tumor_C1 vs. Tumor_C2: r = 0.147) **(F)** gene signatures across tumor subpopulations. **(G)** AUCell score of the Hallmark E2F Targets pathway shows strong enrichment in tumor_C2. Effect sizes: Tumor_C0 vs. Tumor_C1: r = 0.209, Tumor_C0 vs. Tumor_C2: r = 0.649, Tumor_C1 vs. Tumor_C2: r = 0.652 **(H)** AUCell score of immunogenic cell death-related genes indicates increased immunogenicity in tumor_C2. Effect sizes: Tumor_C0 vs. Tumor_C1: r = 0.022, Tumor_C0 vs. Tumor_C2: r = 0.145, Tumor_C1 vs. Tumor_C2: r = 0.139. Data are shown as violin plots with embedded box plots (mean ± SD). Statistical comparisons performed using two-sided Wilcoxon rank-sum test with FDR correction (Benjamini-Hochberg method) for multiple pathway comparisons. Effect sizes reported as r (r = |Z|/√N, where Z is the Wilcoxon Z-statistic and N is total sample size). *q < 0.05, **q < 0.01, ***q < 0.001 (FDR-adjusted q-values). Sample sizes for each comparison are indicated in individual panel captions.

To explore the developmental hierarchy and functional states of HCC tumor subpopulations, we applied pseudotime and CytoTRACE analysis to the tumor cell clusters. Monocle pseudotime analysis revealed a gradient of differentiation potential, with Tumor_C2 displaying the highest developmental immaturity, followed by Tumor_C1 and Tumor_C0 (Figure 3A). This pattern was corroborated by CytoTRACE, which positioned Tumor_C2 at an earlier developmental trajectory compared to the other clusters (Figure 3B). Using CytoTRACE2, an interpretable AI method for predicting cellular potency and absolute developmental potential from single-cell RNA-sequencing data, we performed quantitative assessment of stemness potential across different tumor cell clusters. The CytoTRACE2 analysis demonstrated significantly higher potency scores in Tumor_C2 compared to other clusters, consistent with a less differentiated, more stem-like cellular state (Figure 3C).

To further characterize the functional differences among tumor clusters, we performed AUCell scoring on tumor cell subpopulations. Tumor_C2 exhibited markedly elevated scores in tumor stemness, proliferation, pro-metastatic signaling, and E2F target activation relative to Tumor_C0 and Tumor_C1 (Figures 3D–G). Notably, Tumor_C2 also showed higher levels of immunogenic cell death (Figure 3H), suggesting potential immunogenic features despite its aggressive phenotype. These results highlight Tumor_C2 as a poorly differentiated, highly proliferative, and potentially immunogenic subpopulation within HCC.

### 3.4 *H2AFZ* is essential for hepatocellular carcinoma cell proliferation and invasion

**FIGURE 4:**
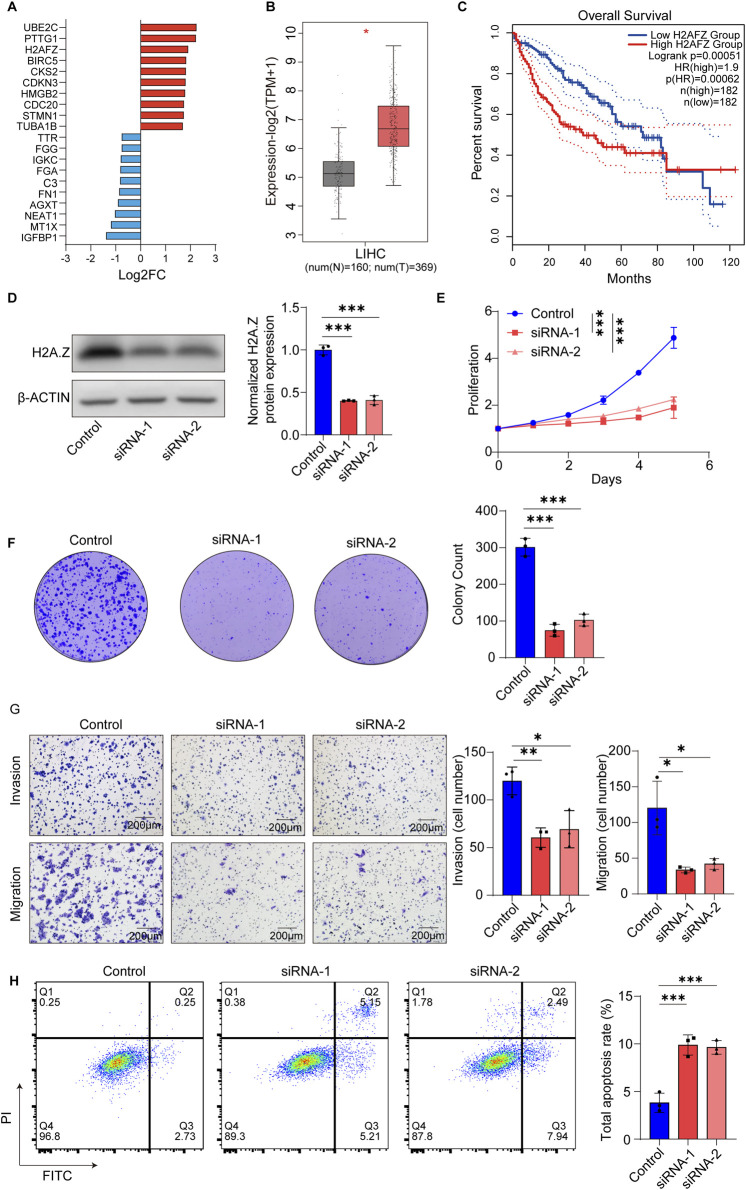
*H2AFZ* promotes proliferation, survival, and migration of HCC cell line SNU-449. **(A)** Bar plot showing top differentially expressed genes in the proliferative tumor_C2 cluster compared with tumor_C0 and tumor_C1, upregulated (red) and downregulated (blue), highlighting upregulation of *H2AFZ*. The x-axis indicates the log2 fold change (Log2FC). **(B)** *H2AFZ* expression in normal (N, n = 160) and tumor (T, n = 369) liver tissues from TCGA-LIHC dataset (TPM+1, log2 transformed). **(C)** Kaplan-Meier analysis showing that high *H2AFZ* expression correlates with poorer OS in HCC patients. **(D)** Representative Western blot images showing H2A.Z and β-actin in SNU-449 cells. **(E)** Cell proliferation curves of control and *H2AFZ*-knockdown cells (*H2AFZ* siRNA-1 and siRNA-2) over 5 days. **(F)** Representative images and quantification of colony formation assay showing reduced clonogenicity upon *H2AFZ* silencing. **(G)** Transwell invasion and migration assays reveal significantly impaired motility in *H2AFZ*-depleted cells; scale bar, 200 μm. **(H)** Flow cytometry-based apoptosis assay using Annexin V/PI staining shows increased apoptosis in knockdown groups. Data are presented as mean ± SD from three independent experiments. Statistical analyses: two-way ANOVA for proliferation, unpaired t-test for colony formation, migration, invasion and apoptosis assays (t statistic reported, effect size reported as Cohen’s **(D)**; log-rank test for survival analysis. *p < 0.05, **p < 0.01, ***p < 0.001.

To identify proliferation-associated tumor cell subpopulations in HCC, we first analyzed cell-type specific transcriptomic profiles and identified *H2AFZ* as one of the top upregulated genes in tumor_C2 cells (Figure 4A). Expression analysis based on the TCGA-LIHC dataset confirmed that *H2AFZ* expression was significantly elevated in tumor tissues compared with adjacent normal tissues (Figure 4B), and high *H2AFZ* expression was associated with poorer OS (Figure 4C), suggesting a potential oncogenic role.

To further characterize its function, we performed knockdown experiments using two independent siRNAs targeting *H2AFZ* in HCC cell line SNU-449 and Huh7 (Figure 4D; Supplementary Figure S2A). *H2AFZ* silencing markedly suppressed HCC cell line SNU-449 proliferation (Figure 4E) and colony formation capacity (control vs. siRNA-1, t = 13.52, Cohen’s d = 11.04, 95% CI = [-273.2, −180.1], *p* = 0.0004; control vs. siRNA-2, t = 11.85, Cohen’s d = 9.67, 95% CI = [-245.2, −152.1]; *p* = 0.0003) (Figure 4F). Transwell assays revealed that *H2AFZ* depletion significantly reduced both invasive (control vs. siRNA-1, t = 4.444, Cohen’s d = 3.63, 95% CI = [-278.4, −64.3], *p* = 0.0113; control vs. siRNA-2, t = 4.952, Cohen’s d = 4.04, 95% CI = [-346.1, −97.41]; *p* = 0.0078) and migratory (control vs. siRNA-1, t = 9.002, Cohen’s d = 7.35, 95% CI = [-277.4, −146.6], *p* = 0.0008; control vs. siRNA-2, t = 10.71, Cohen’s d = 8.74, 95% CI = [-354.6, −208.6]; *p* = 0.0004) capabilities of HCC cells (Figure 4G), implicating its role in metastatic progression. Flow cytometry analysis demonstrated that *H2AFZ* knockdown significantly increased apoptotic cell populations (control vs. siRNA-1, t = 7.178, Cohen’s d = −5.86, 95% CI = [3.722, 8.418], *p* = 0.002; control vs. siRNA-2, t = 8.098, Cohen’s d = −6.61, 95% CI = [3.829, 7.824]; *p* = 0.0013) (Figure 4H), further indicating that *H2AFZ* promotes HCC cell growth by preventing apoptosis. In line with these findings, another HCC cell line Huh7 also showed inhibited cell proliferation and colony formation capacity, suppressed migratory and invision ability, increased apoptotic cell populations (Supplementary Figure S2B–E).

### 3.5 Single-cell transcriptome analysis reveals distinct T cell functional states

**FIGURE 5:**
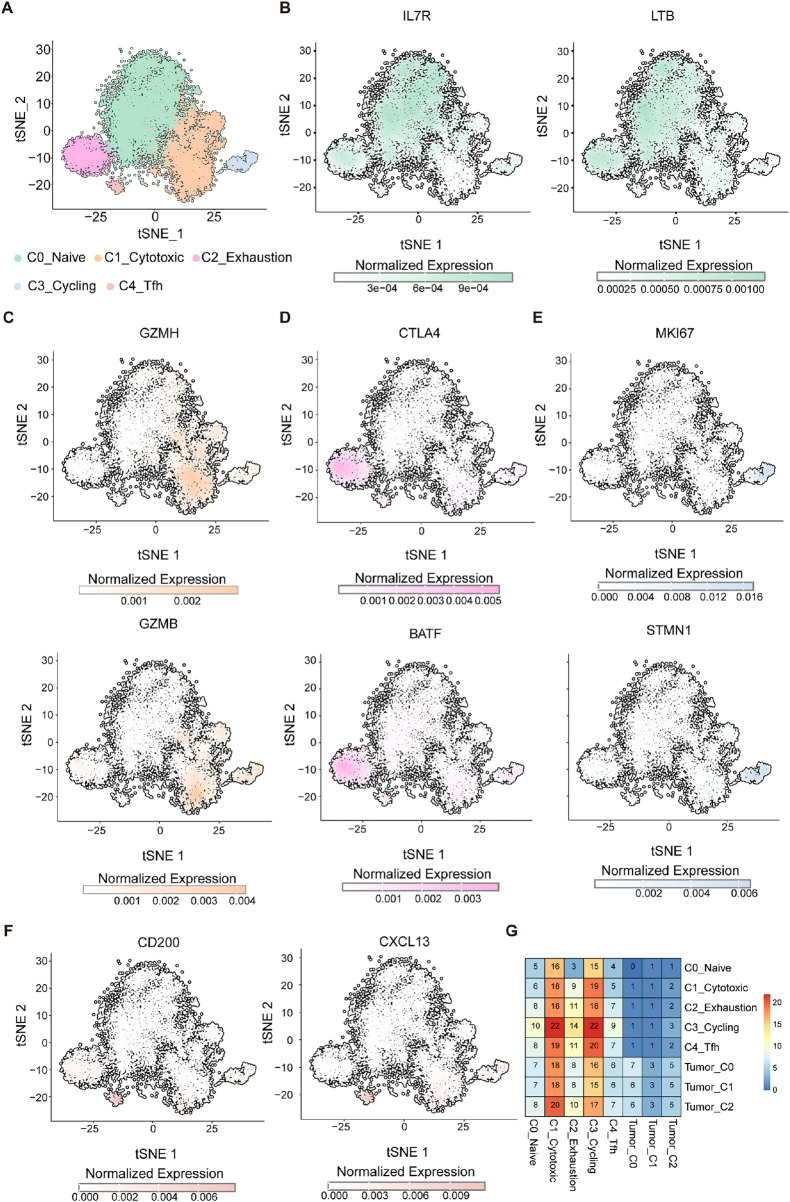
Single-cell transcriptome analysis identifies functionally distinct T cell populations. **(A)** UMAP visualization of CD8^+^ T cells grouped into five transcriptionally distinct clusters: C0-Naïve (pale green), C1-Cytotoxic (light orange), C2-Exhaustion (light pink), C3-Cycling (light blue), and C4-Tfh (soft red). **(B–F)** t-SNE plots showing normalized expression of representative marker genes across the CD8^+^ T cell landscape. **(B)** Naïve markers: IL7R and LTB. **(C)** Cytotoxic markers: GZMH and GZMB. **(D)** Exhaustion markers: CTLA4 and BATF. **(E)** Proliferation markers: MKI67 and STMN1. **(F)** Tfh-like markers: CD200 and CXCL13. Expression values are color-coded by intensity (grey to colored scale), reflecting normalized transcript abundance per cell. **(G)** Heatmap showing the interaction strength between T-cell subpopulations and tumor cell clusters. The color scale represents interaction intensity, with red indicating stronger interactions and blue indicating weaker interactions.

To gain deeper insight into the immune landscape, particularly the role of T cells in the context of the immunologically active Tumor_C2 subpopulation, we performed T cells cluster analysis (Zhang et al., 2018). TSNE dimensionality reduction revealed five distinct cell clusters, naive T cells (C0_Naive), cytotoxic T cells (C1_Cytotoxic), exhausted T cells (C2_Exhaustion), cycling T cells (C3_Cycling), and T follicular helper cells (C4_Tfh) (Figure 5A). To more accurately define these subpopulations, we visualized the expression of representative marker genes on the t-SNE plot and bubble plot (Figures 5B–F; Supplementary Figure S3). Naïve T cells (C0_Naive) were enriched for IL7R and LTB (Figure 5B), consistent with their quiescent and survival-supporting phenotype. The cytotoxic effector molecules GZMH and GZMB were predominantly expressed in Cytotoxic T cells (C1_Cytotoxic), reflecting their potent cytolytic capacity (Figure 5C). The exhausted subset (C2_Exhaustion) expressed elevated levels of immune checkpoint regulators such as CTLA4 and transcription factor BATF (Figure 5D), indicative of a dysfunctional state. Cycling T cells (C3_Cycling) demonstrated high proliferative activity as evidenced by MKI67 and STMN1 expression (Figure 5E). Lastly, the C4_Tfh cluster showed specific expressions of CD200 and CXCL13 (Figure 5F), suggestive of a specialized function in lymphoid tissue organization and B cell help.

To evaluate the relationship between T-cell subpopulations within tumor-infiltrating lymphocytes (TILs) and different tumor cell subgroups, we analyzed communication patterns across all tumor cell and T-cell subpopulations. A strong correlation was observed between the C3_Cycling and C2_Exhaustion T-cell subpopulations and the Tumor_C2 cluster (Figure 5G), suggesting a potential interplay among these subsets. The Tumor_C2 subgroup may modulate the formation of an immunosuppressive microenvironment through interactions with proliferating and exhausted T cells, indicating a possible mechanism of immune escape. These findings offer valuable insights into potential therapeutic strategies targeting specific immune subpopulations. For instance, treatments capable of reversing T-cell exhaustion, such as immune checkpoint inhibitors, may be particularly effective in this context by reinvigorating exhausted T cells and enhancing their anti-tumor activity. Furthermore, a deeper understanding of the interplay between proliferating and exhausted T cells within the tumor microenvironment could aid in the development of immunotherapies aimed at overcoming tumor-induced immune tolerance.

### 3.6 Prognostic model stratifies patient risk and predicts clinical outcomes

**FIGURE 6:**
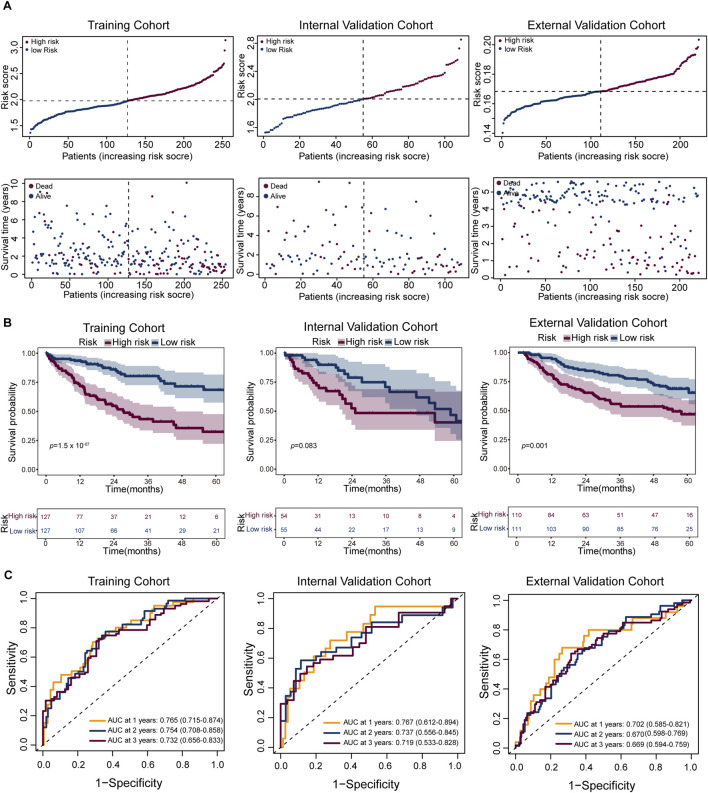
Performance of the prognostic risk model across cohorts. **(A)** Risk score distribution (top) and corresponding survival status (bottom) for patients in the training (left), internal validation (middle), and external validation (right) cohorts. Patients are ranked by increasing risk score; dashed lines indicate the median cutoff used to stratify high- and low-risk groups. **(B)** Kaplan–Meier survival analysis comparing overall survival (OS) between high-risk (red) and low-risk (blue) groups in the three cohorts. Shaded areas represent 95% confidence intervals. The number of patients at risk is shown below each plot. **(C)** Receiver operating characteristic (ROC) curves evaluated model performance for 1-, 2-, and 3-year survival predictions, with respective AUC values indicated.

In recent years, machine learning algorithms have been widely applied to the identification of prognostic biomarkers and the construction of predictive models in cancer (Yuan et al., 2025). We constructed a prognostic model based on the signature gene set of the Tumor C2 subpopulation (Supplementary Table S1), and evaluated its performance in the training cohort, internal validation cohort, and external validation cohort. The distribution of risk scores showed a clear separation between the two groups (Figure 6A). Kaplan-Meier survival analysis revealed that high-risk patients had significantly worse overall survival (OS) in the training cohort (log-rank test; n = 254; HR = 0.32, 95% CI: 0.2–0.5; *p* = 1.51 × 10^−7^), and this trend remained consistent in the internal validation cohort (log-rank test; n = 109; HR = 0.58, 95% CI: 0.31–1.08; *p* = 0.083) and external validation cohort (log-rank test; n = 221; HR = 0.49, 95% CI: 0.32–0.76; *p* = 0.001) (Figure 6B). Time-dependent ROC analysis supported the predictive performance of the model, with AUCs of 0.765 (95% CI: 0.715–0.874), 0.754 (95% CI: 0.708–0.858), and 0.732 (95% CI: 0.656–0.833) for 1-, 2-, and 3-year survival prediction in the training cohort (n = 254); 0.767 (95% CI: 0.621–0.894), 0.737 (95% CI: 0.556–0.845), and 0.719 (95% CI: 0.533–0.828) in the internal validation cohort (n = 109); and 0.702 (95% CI: 0.585–0.821), 0.670 (95% CI: 0.598–0.769), and 0.669 (95% CI: 0.594–0.759) in the external validation cohort (n = 221), respectively (Figure 6C). These findings suggest that the model can effectively stratify patient risk and robustly predict survival outcomes across independent datasets.

## 4 Discussion

HCC is one of prototypical immunogenic and heterogeneous solid tumor (Puram et al., 2017; Zhang and Zhang, 2020; Peng et al., 2019), characterized by complex cellular interactions within its TME. The substantial intra-tumoral heterogeneity remains a major barrier to effective treatment. Thus, elucidating the cellular composition and underlying mechanisms is crucial for improving prognosis and guiding therapy.

In this study, leveraging integrative scRNA-seq analysis of HCC from three independent datasets, generating a comprehensive single-cell atlas. Notably, we identified three transcriptionally distinct tumor cell subtypes: Tumor_C0 (ALDOB^high^ and ARG1^high^), Tumor_C1 (S100A6^high^ and S100A11^high^) and Tumor_C2 (STMN1^high^ and HMGB2^high^). Tumor_C2 cells exhibited elevated proliferative capacity and stemness features. We further validated *H2AFZ* as a biomarker of this subtype. Importantly, the consistent presence of Tumor_C2 across all patients suggests shared therapeutic vulnerabilities that may transcend inter-patient heterogeneity. Moreover, Tumor_C2 showed enhanced interaction with T-cell subsets, particularly C3_Cycling and C2_Exhaustion, indicating active tumor–immune crosstalk.

ARG1, highly expressed in Tumor_C0, has been used as a diagnostic marker for well-differentiated HCC (Clark et al., 2017; Atta, 2021). However, reduced ARG1 expression is associated with poor prognosis (Shigematsu et al., 2022). Regarding the Tumor_C1 subtype, its high expression of S100A6 and S100A11-calcium binding proteins known to promote tumor cell migration and invasion (Gross et al., 2014; Nedjadi et al., 2009), suggests an association with EMT and metastatic potential, indicating this subtype may represent a therapeutically targetable population.

However, trajectory analysis using pseudotime and CytoTRACE placed Tumor_C2 at the apex of the developmental hierarchy, consistent with a progenitor-like origin or dedifferentiated hepatocyte phenotype. This suggests that Tumor_C2 may represent a cancer stem-like population capable of generating more differentiated Tumor_C1 and Tumor_C0 subtypes. This aligns with the cancer stem cell (CSC) model (Lee et al., 2022), which proposes that targeting stem-like populations may be more effective in limiting tumor progression and metastasis.

Among the differentially expressed genes in Tumor_C2, *H2AFZ*, encoding the histone variant H2A.Z, was significantly upregulated. Elevated *H2AFZ* expression has been associated with genomic instability, chromatin accessibility, and oncogenic transcription programs (Liu et al., 2022; Long et al., 2020). In other cancers, *H2AFZ* promotes proliferation, inhibits senescence, and reduces therapy sensitivity (Jostes et al., 2024; Avila-Lopez et al., 2021). Our functional assays confirmed *H2AFZ*’s role in driving proliferation, invasion, colony formation, and resistance to apoptosis in HCC cells. Its knockdown induced G1-phase arrest and apoptosis, suggesting it supports tumor cell survival via chromatin remodeling. While these *in vitro* findings suggest that *H2AFZ* may represent a candidate therapeutic target for aggressive HCC subtypes, several important considerations warrant further investigation. First, the therapeutic potential of *H2AFZ* requires validation in appropriate *in vivo* models to confirm anti-tumor efficacy and assess potential toxicity to normal tissues. Second, as a histone variant involved in fundamental chromatin biology, the druggability of *H2AFZ* presents technical challenges that need to be addressed, including the development of specific inhibitors and evaluation of off-target effects on normal cellular processes. Additionally, given the essential role of *H2A.Z* in normal cell physiology, careful assessment of the therapeutic window and potential adverse effects will be critical for any future drug development efforts targeting this pathway.

In parallel, we further dissected the immune landscape of HCC, focusing on T cells, the main effectors of immune surveillance (Wu et al., 2022; Silva-Santos et al., 2019). Five transcriptionally distinct T cell clusters were identified, representing naive, cytotoxic, exhausted, and proliferative states. Importantly, exhausted (C2_Exhaustion) and cycling (C3_Cycling) T cell subsets showed strong interaction with tumor_C2, likely reflecting chronic antigen exposure by highly proliferative tumor cells driving T cell exhaustion.

Interestingly, Tumor_C2 not only exhibited proliferative and stem-like features but also displayed a high score for immunogenic cell death (ICD) gene signatures, such as calreticulin exposure and ATP release. This apparent paradox—coexistence of immunogenic potential and immune suppression—suggests a dynamic interplay where initial immune activation is rapidly followed by exhaustion and dysfunction, a phenomenon frequently observed in advanced HCC (Hegde and Chen, 2020; Zhou et al., 2017; Galluzzi et al., 2017). These data support the rationale for combination therapies that both reverse T cell exhaustion (e.g., PD-1 blockade) and enhance tumor ICD (e.g., radiation, oncolytic viruses).

To bridge molecular discovery with clinical relevance, we developed a prognostic risk model based on transcriptional signatures derived from tumor subsets. The model demonstrated robust predictive capacity of the gene set of tumor_C2 for overall survival in training and validation cohorts, underscoring its potential as a prognostic stratification tool.

Although the study offers novel insights, several limitations remain. First, the cross-sectional nature of scRNA-seq limits the ability to track lineage evolution dynamically. Second, the spatial context of tumor–immune interactions require validation using spatial transcriptomics or multiplex immunofluorescence. Third, while *H2AFZ* is functionally validated *in vitro*, it’s *in vivo* relevance, potential therapeutic index, and interaction with immune responses warrant further investigation.

In conclusion, this study delineates a comprehensive single-cell map of HCC, identifying a key malignant subpopulation (Tumor_C2) with stem-like, proliferative, and immunomodulatory properties. The histone variant H2A.Z(encoded by *H2AFZ*) emerges as a central oncogenic driver within this context. The observed coupling between tumor cell state and T cell dysfunction underscores the need for integrative therapeutic strategies targeting both cancer-intrinsic and immune-mediated pathways.

## Data Availability

The datasets presented in this study can be found in online repositories. The names of the repository/repositories and accession number(s) can be found in the article/Supplementary Material.

## Supplementary material

SUPPLEMENTARY FIGURE S1 Single-cell transcriptomic analysis across patients. **(A)** t-SNE visualization of single-cell RNA sequencing data from tumor samples across multiple patients. **(B)** Heatmap of inferred copy number variation (CNV) profiles across tumor cells (pink) and T cells (green) from all patients. Each column represents a chromosome (1-22), and rows correspond to single cells. Gains and losses are represented in red and blue, respectively. **(C)** Bubble plot of representative marker gene expression across three tumor cell subclusters (Tumor_C1, Tumor_C2, Tumor_C3). Dot size represents the percentage of cells expressing the gene, and color intensity indicates average expression levels.

SUPPLEMENTARY FIGURE S2
*H2AFZ* promotes proliferation, survival, and migration of HCC cell line Huh7. **(A)** Representative Western blot images showing H2A.Z and β-actin in Huh7 cells. **(B)** Cell proliferation curves of control and *H2AFZ*-knockdown cells. **(C)** Representative images and quantification of colony formation assay showing reduced clonogenicity upon *H2AFZ* silencing. **(D)** Transwell invasion and migration assays reveal significantly impaired motility in *H2AFZ*-depleted cells; scale bar, 200 μm. **(E)** Flow cytometry-based apoptosis assay shows increased apoptosis in knockdown groups. Data are presented as mean ± SD from three independent experiments. Statistical analyses: two-way ANOVA for proliferation, unpaired t-test for colony formation, migration, invasion and apoptosis assays. **p* < 0.05, ***p* < 0.01, ****p* < 0.001.

SUPPLEMENTARY FIGURE S3 Bubble plot of representative marker gene expression across five T cell subclusters. Bubble size represents the percentage of cells expressing the gene, and color intensity indicates average expression levels.

## Abbreviations

GEO, Gene Expression Omnibus; HCC, hepatocellular carcinoma; LASSO, Least Absolute Shrinkage and Selection Operator; DFS, disease-free survival; scRNA-seq, single-cell RNA sequencing; TME, Tumor microenvironment; TIME, tumor immunomicroenvironment; TCGA, The Cancer Genome Atlas; LIHC, liver hepatocellular carcinoma.
