# Supplementary figures and images for: Single-cell transcriptomics identifies an H2AFZ-driven proliferative tumor subpopulation associated with poor prognosis in hepatocellular carcinoma
Source: Front Mol Biosci. 2025 Oct 8;12:1655705. doi: 10.3389/fmolb.2025.1655705 (PMC12540112; doi:10.3389/fmolb.2025.1655705)

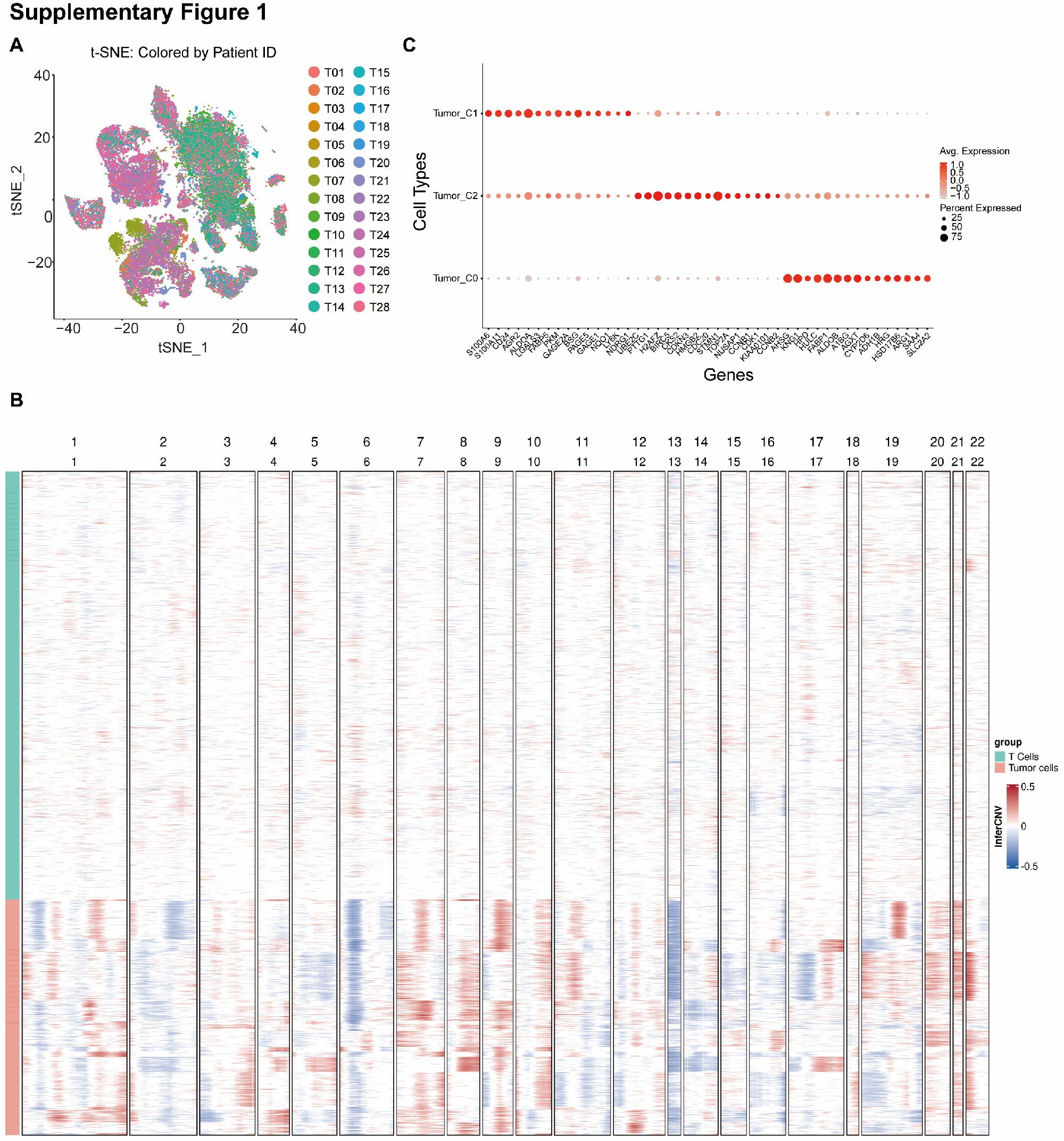

Supplement: Supplementary file 1 [file Image1.tiff]

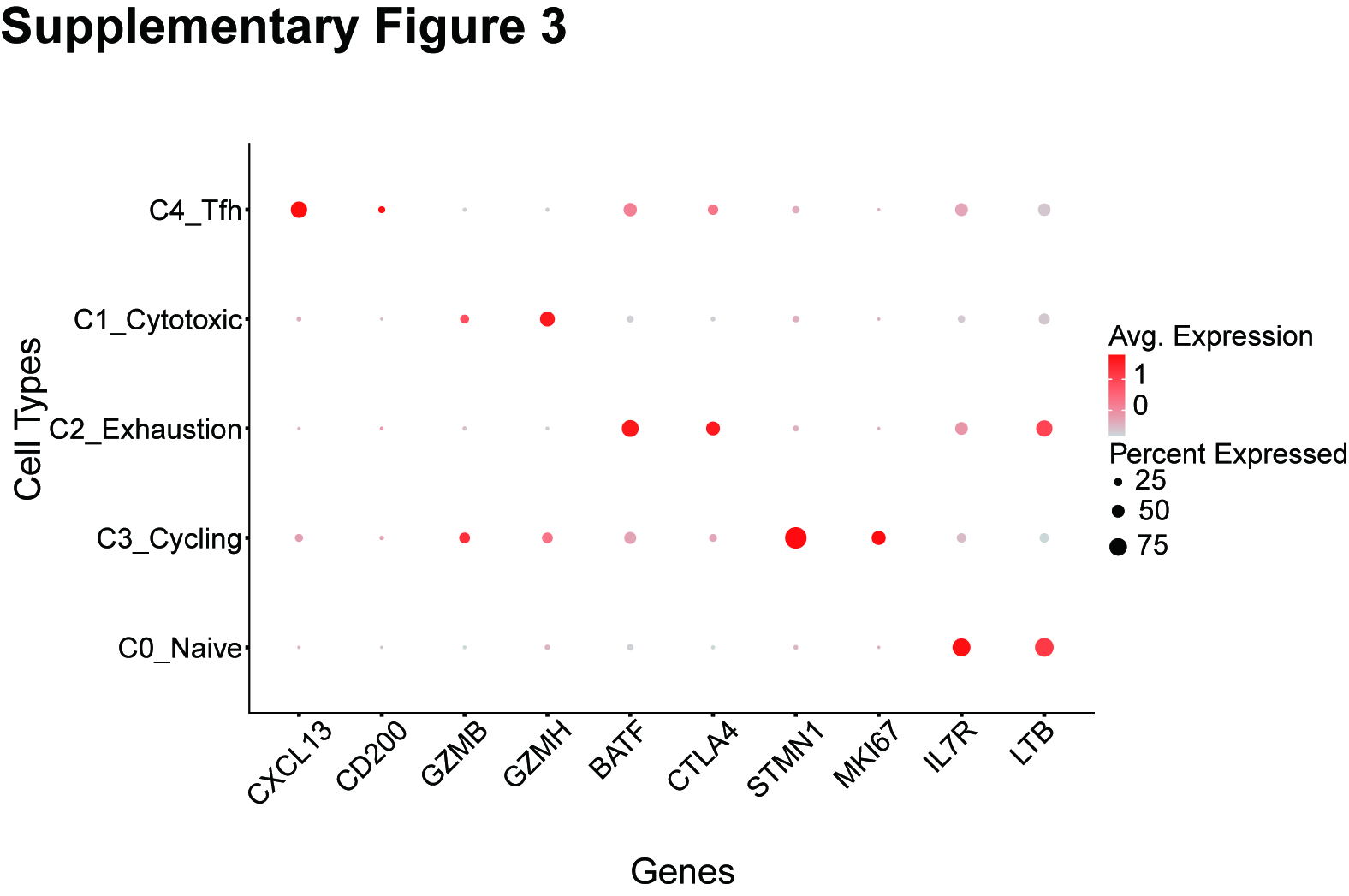

Supplement: Supplementary file 2 [file Image3.tif]

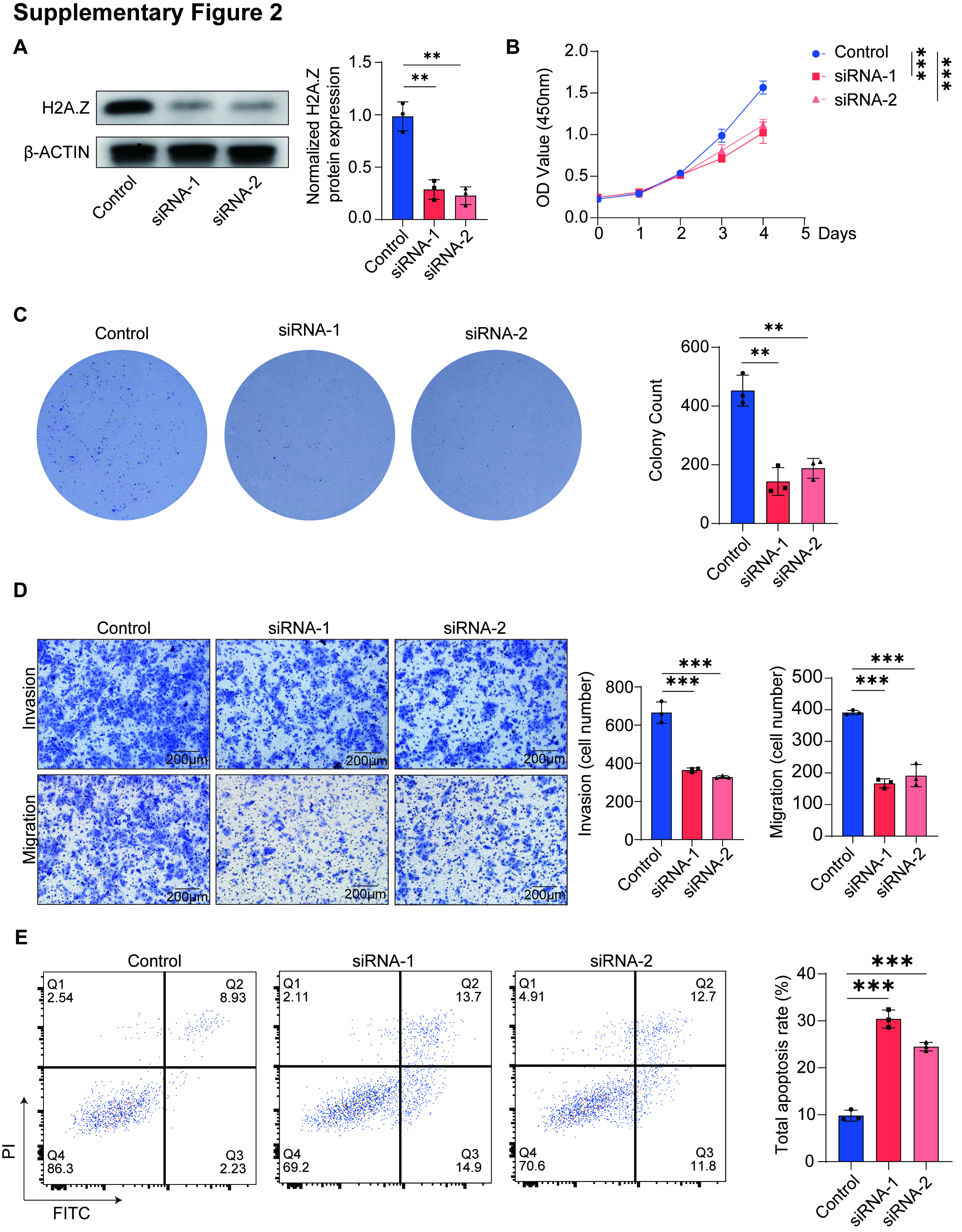

Supplement: Supplementary file 3 [file Image2.tif]
